# Removing Noise From Pyrosequenced Amplicons

**DOI:** 10.1186/1471-2105-12-38

**Published:** 2011-01-28

**Authors:** Christopher Quince, Anders Lanzen, Russell J Davenport, Peter J Turnbaugh

**Affiliations:** 1 Department of Civil Engineering, University of Glasgow, Rankine Building, Oakfield Avenue, Glasgow G12 8LT, UK; 2 Department of Biology, Centre for Geobiology, University of Bergen, Bergen, Norway; 3 School of Civil Engineering and Geosciences, University of Newcastle upon Tyne, Newcastle upon Tyne NE1 7RU, UK; 4 FAS Center for Systems Biology, Harvard University, Cambridge, MA 02138, USA

## Abstract

**Background:**

In many environmental genomics applications a homologous region of DNA from a diverse sample is first amplified by PCR and then sequenced. The next generation sequencing technology, 454 pyrosequencing, has allowed much larger read numbers from PCR amplicons than ever before. This has revolutionised the study of microbial diversity as it is now possible to sequence a substantial fraction of the 16S rRNA genes in a community. However, there is a growing realisation that because of the large read numbers and the lack of consensus sequences it is vital to distinguish noise from true sequence diversity in this data. Otherwise this leads to inflated estimates of the number of types or operational taxonomic units (OTUs) present. Three sources of error are important: sequencing error, PCR single base substitutions and PCR chimeras. We present AmpliconNoise, a development of the PyroNoise algorithm that is capable of separately removing 454 sequencing errors and PCR single base errors. We also introduce a novel chimera removal program, Perseus, that exploits the sequence abundances associated with pyrosequencing data. We use data sets where samples of known diversity have been amplified and sequenced to quantify the effect of each of the sources of error on OTU inflation and to validate these algorithms.

**Results:**

AmpliconNoise outperforms alternative algorithms substantially reducing per base error rates for both the GS FLX and latest Titanium protocol. All three sources of error lead to inflation of diversity estimates. In particular, chimera formation has a hitherto unrealised importance which varies according to amplification protocol. We show that AmpliconNoise allows accurate estimates of OTU number. Just as importantly AmpliconNoise generates the right OTUs even at low sequence differences. We demonstrate that Perseus has very high sensitivity, able to find 99% of chimeras, which is critical when these are present at high frequencies.

**Conclusions:**

AmpliconNoise followed by Perseus is a very effective pipeline for the removal of noise. In addition the principles behind the algorithms, the inference of true sequences using Expectation-Maximization (EM), and the treatment of chimera detection as a classification or 'supervised learning' problem, will be equally applicable to new sequencing technologies as they appear.

## Background

Next generation sequencing has revolutionised many areas of biology by providing a cheaper and faster alternative to Sanger sequencing. One technology that is finding many applications, for example in *de novo *genome sequencing, or diversity studies of regions of DNA that have been amplified by PCR, is 454 Pyrosequencing [1]. It is this latter application of 454 to the sequencing of PCR products or amplicons that we will focus on here. 454 Pyrosequencing generates large numbers of reads, 400,000 in the original GS FLX implementation increasing to 800,000 with Titanium reagents, which are long compared to other pyrosequencing platforms, 250 bp for GS FLX increasing to around 500 bp for Titanium. This makes it ideal for high resolution studies of the sequences and their relative frequencies in relatively long PCR products. The method is to simply sequence the diverse amplicon sample without cloning individual sequences. This has many applications for instance in viral population dynamics [2], or characterising microbial communities through amplification of 16S rRNA genes [3].

Per base error rates from 454 pyrosequencing are comparable to those from Sanger sequencing [4] but without cloning resequencing is impossible. In addition, the large read numbers obtainable mean that the absolute number of noisy reads is substantial. Consequently, it is critical to distinguish true diversity in the sample from noise introduced by the experimental procedure. This is particularly true if we want to calculate the absolute number of different sequences, or clusters of sequences, present. This is effectively the problem in microbial diversity estimation, where sequences are clustered into Operational Taxonomic Units (OTUs) that proxy for traditional taxa and we are interested in estimating the number of such OTUs in a community. It has already been noted that noise in 454 amplicon sequencing leads to inflated estimates of OTU number [5,6]. This is important because surprisingly large OTU numbers together with a bias towards rare taxa were observed in the first studies of pyrosequenced 16S rRNA genes [3,7]. This preponderance of rare taxa has been termed the 'rare biosphere'. The spurious OTUs generated by noise generally have low frequencies consequently noise may explain both the high OTU numbers and the bias towards low abundances reported. Development of effective noise removal algorithms is therefore a matter of urgency in the exploration of microbial diversity.

PyroNoise is a relatively sophisticated algorithm that reconstructs the true sequences and frequencies in the sample prior to OTU construction using a mixture model [5]. It is based on clustering flowgrams rather than sequences which allows 454 errors to be modelled naturally. Using this approach it is possible to account for two facts: firstly that sequences with errors are likely to be rare and secondly that they should be similiar to a true abundant sequence. The mixture model approach allows this to be done in a very natural way by fitting noise distributions around each proposed true sequence. The drawback is that an iterative and hence computationally costly algorithm must be used.

Two sources of error need to be considered in pyrosequenced amplicons. Those from the pyrosequencing itself and those introduced by the PCR amplification. The original implementation of PyroNoise simultaneously removed both sources of errors. Consequently it was necessary to align flowgrams. Here we present a new approach, AmpliconNoise, which couples a fast flowgram clustering step without alignment, still called PyroNoise, to a sequence based clustering, SeqNoise, which does perform alignments. The latter explicitly accounts for the differential rates of nucleotide errors in the PCR process, and uses sequence frequencies to inform the clustering process. The result is a more sensitive program than the original PyroNoise achieved at lower computational cost because the fast alignment free flowgram clustering reduces the data set size for the slower sequence clustering. AmpliconNoise has already been used to determine gut microbial diversities [8] and for viral population genetics [2].

Recently another flowgram based denoising algorithm, DeNoiser, has been developed [9]. This was motivated by the original PyroNoise and uses the same flowgram alignments but incorporates several modifications to increase speed. It begins by finding unique sequences, orders them by frequency, and then starting with the most abundant maps the other reads onto these 'centroids' if their distance to the centroid is smaller than some threshold. The distance used is the same flowgram based measure as in the original PyroNoise. It is therefore a greedy agglomerative clustering rather than iterative. This reduces the computational costs of the algorithm but misassignment of reads when the true sequences are similar, may result in a loss of ability to accurately reconstruct OTUs. An even faster approach is to perform the same centroid based clustering using sequence rather than flowgram based distances, this is referred to as single-linkage preclustering (SLP) by Huse et al. [10], and a similar strategy is adopted in the PyroTagger program [11]. In this paper we will describe AmpliconNoise fully for the first time and compare to the original PyroNoise algorithm, DeNoiser, and SLP in terms of ability to remove noise and allow accurate OTU construction. We will use 454 pyrosequencing data from known sequences for these evaluations including both standard GS FLX and newer Titanium data.

SeqNoise accounts for PCR single base errors but the PCR process can also introduce sequences that are composed of two or more true sequences, so called 'chimeras'. These generate sequences that are quite different from either parent and so can not be removed by clustering. Chimeras are generated when incomplete extension occurs during the PCR process and the resulting fragment effectively acts as the primer in the next round of PCR. Existing algorithms for removing chimeras were developed for full length clone sequences and lack the sensitivity for short pyrosequencing reads [12,13]. The program ChimeraSlayer is to our knowledge the only current chimera checker capable of handling 454 reads effectively. ChimeraSlayer requires a reference data set of sequences that are known to be non-chimeric [14]. We introduce a new algorithm, Perseus, based on two novel principles for chimera removal: firstly because the parents of any chimera must experience at least one more round of PCR than the chimera then we can search amongst all those sequences of equal or greater abundance than the chimera for possible parents; secondly that chimera removal should, with suitable training data sets, be treated as a problem in classification or 'supervised learning'. Adopting these principles Perseus has the sensitivity to remove chimeras from 250 bp GS FLX reads with the advantage of not requiring a set of good reference sequences.

We will now demonstrate that AmpliconNoise followed by Perseus is capable of removing the vast majority of erroneous reads from 454 pyrosequencing data, reducing overall error rates, and thereby allowing accurate OTU construction and microbial diversity estimation.

## Methods

### Test Data Sets

To test the noise removal algorithms we used eight previously published test data sets and one hitherto unpublished. These were all generated by preparing mixtures of known DNA sequences in known concentrations and amplifying and pyrosequencing. For the published data sets the standard GS FLX protocol was used but specifically for this study we generated a further data set with the most recent Titanium reagents. Three different 16S rRNA regions were amplified in all cases with a standard Taq polymerase, the V2 region [8], the V5 region [5], and for the Titanium data V4-V5. The mixtures consisted either of 16S rRNA clones in the case of the V5 and V4-V5 data sets or DNA extracted from 67 separate isolated organisms for V2. Two V5 data sets were prepared: one 'Divergent' data set comprising 23 clones that differed at a least 7% of nucleotide positions mixed in equal proportions, facilitating the unambiguous mapping of each read to a known clone, and one 'Artificial Community' where some clones differed by just a single nucleotide and concentrations varied by two orders of magnitude mimicking a natural community. The V2 'Mock Communities' were similarly split between three replicates where the extracted DNA was mixed in equal proportions (Even1, Even2, Even3) and three where it was mixed unevenly (Uneven1, Uneven2, Uneven3). Full details of these test data sets are available from the original publications [5,8].

The Titanium data was generated by pyrosequencing a mixture of 91 full length 16S rRNA clones obtained from an Arctic soil sample. These clones were independently Sanger sequenced although only 89 sequences could actually be recovered. Consequently the results presented here will be a lower bound on accuracy with a few sequences falsely categorised as errors that should be in the sample. Since this will apply equally to all the tested algorithms our ability to compare between them is not affected. The mixture contained each clone in equal abundance. This DNA mixture was then pyrosequenced following amplification with 16S rRNA primers that also had a tag (AGTGCGTA) and the standard Titanium A and B adaptors attached. The primers used were both degenerate, F515 (GTGNCAGCMGCCGCGGTAA) and R926 (CCGYCAATTYMTTTRAGTTT). Sequencing was forward from F515 so as to capture the V4 and most of the V5 region with a 400-500 bp Titanium read.

Table 1 summarises each of the nine data sets used in this study and includes the number of reads obtained for each.

**Table 1:** The eight GS FLX and one Titanium test data sets used in this study

Name	Type	Abundances	16S region	**Read no**.	**Filtered no**.	**DeNoiser no**.
Divergent	23 clones	Even	V5	57,902	35,190	42,052
Artificial	90 clones	Uneven	V5	46,249	31,867	37,903

Mock Communities						

Even1	67 genomic	Even	V2	63,780	53,771	55,398
Even2	DNA isolates	Even	V2	53,763	45,178	46,294
Even3	DNA isolates	Even	V2	67,182	54,153	55,797
Uneven1	DNA isolates	Uneven	V2	54,099	44,926	46,837
Uneven2	DNA isolates	Uneven	V2	51,439	44,176	44,880
Uneven3	DNA isolates	Uneven	V2	60,976	50,931	53,225

Titanium	91 clones	Even	V4 - V5	62,873	25,438	21,477

The 16S rRNA region amplified, and whether references were mixed in equal, 'Even', or varying, 'Uneven' proportions are summarised together with the read number, following filtering and after the QIIME filtering used by the DeNoiser algorithm. All data sets were generated by GS FLX except that denoted Titanium.

### Origins of Pyrosequencing Noise

**Figure 1:**
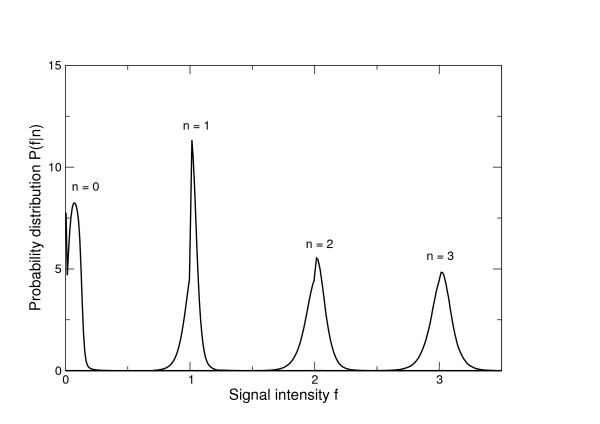
**Flowgram signal intensity distributions**. Probability distributions of observed signal intensities at different homopolymer lengths for the 'Even' V2 Mock Communities. The homopolymer length is shown above the mode of the distribution.

Pyrosequencing is a sequencing by synthesis method. Single molecules of DNA are attached to beads and subjected to emulsion PCR to generate multiple identical copies. The beads are then localised into separate wells on plates that contain hundreds of thousands of such wells. These wells contain the enzymes and substrates necessary for DNA synthesis such that if synthesis occurs light is emitted. Each base in turn (generally in the order ATGC so that the signal translates by TACG) is then washed across the plate. If a well contains a DNA molecule where the next unpaired base is complimentary then a signal is observed. If a homopolymer is present then further synthesis will occur and the signal is increased. Consequently a well should emit at least one signal in each frame of four bases. The pattern of light intensities, or flowgram, emitted by each well can then be used to determine the DNA sequence. The standard base-calling procedure is to round the continuous intensities to integers. Errors occur because the observed light intensities do not perfectly match the homopolymer lengths. Instead, a distribution of light intensities is associated with each length. We will denote this distribution by *P *( *f *|*n*) where the observed signal intensity is *f *and the homopolymer length that generates it *n*. These distributions calculated for the three 'Even' V2 'Mock Communities' are shown in Figure 1. The variance of these distributions increases with length consequently the probability of a miss-call where a signal is rounded to the wrong integer does too. These appear as either insertions or deletions.

### Filtering Noisy Reads

It has previously been shown that some features correlate with noise in reads from 454 data [4,6]. Consequently filtering for those reads can reduce the overall level of noise in the data set. However, substantial noise remains after this process [5]. The purpose of this study is not to evaluate different filtering methods for reads, but rather to address the problem of how to account for this remaining noise. Consequently we adopted a rather strict filtering procedure: first we checked for an exact match to the primer and tag if present, we then used the observation that signal intensities between 0.5-0.7 are associated with noisy reads [4]. We therefore truncated all reads at the first such signal, or any sequence of the four nucleotide flows that failed to give a signal ≥ 0.5, any read where this occurred before flow 360 of the 400 flows in a GS FLX run we removed. For the Titanium reads which have 800 flows we used the same procedure keeping only reads where the first noisy flow occurred on or after 360. In addition, the level of noise in reads increases towards the ends of reads [15], to account for this we removed the last 10% of flows truncating all GS FLX reads at flow 360 and Titanium at 720. The numbers of reads after filtering for each data set are given in Table 1.

### Removing Pyrosequencing Noise

Our original algorithm for noise removal [5], began with the observation that to describe pyrosequencing noise it is natural to use distances defined not with sequences but with the flowgrams. To define these distances we begin by calculating the probability that a given flowgram $\bar{f}=\left(f_{\text{1}},\ldots ,f_{M}\right)$ of length *M *will be generated by a sequence of nucleotides $\bar{S}$ that maps to a perfect flowgram, i.e. one generated without noise, *Ū *(for example the sequence CAAAGTGGG becomes [0,0,1,0:0,3,0,1:1,0,0,3] when nucleotides are revealed in the order TACG). Assuming that each signal is independent then this is simply the product of the individual probabilities of each signal. We then take the negative logarithm of the probability and normalise by the flowgram length *M *:

$$\begin{array}{c} d^{\prime }\left(\bar{f},\bar{U}\right)=-log\left(\prod_{i=1}^{M}P\left(f_{i}\;|\;u_{i}=n\right)\right)/M\\ =\sum_{i=1}^{M}-log\left[P\left(f_{i}\;|\;u_{i}=n\right)\right]/M\\ =\sum_{i=1}^{M}d\left(f_{i}\;|\;u_{i}=n\right)/M, \end{array}$$

to generate a total distance which is simply the sum of the distances for each signal. We used the V2 distributions, Figure 1, to calculate flowgram distances for all the GS FLX data sets but calculated new distributions for the Titanium data.

Pyrosequencing noise removal can be posed as the problem of inferring the true sequences and their abundances in the sample given the observed reads. We begin by defining the likelihood of the observed data. This has a natural interpretation as a mixture model where each component of the mixture is a different true sequence. We assume that each of the *N *flowgrams indexed *i *are distributed as exponentials about the *L *true sequences indexed *j *with a characteristic cluster size *σ_p_*:

(1) $$ℱ\left({\bar{f}}_{i}\;|\;{\bar{S}}_{j}\to {\bar{U}}_{j}\right)=\frac{\exp\left[-d^{\prime }\left({\bar{f}}_{i},{\bar{U}}_{j}\right)\;/\;\sigma_{p}\right]}{\sigma_{p}}$$

The likelihood of the complete data set *D *is then the product of the probabilities that each read was generated from the mixture of sequences with relative frequencies *τ_j_*:

(2) $$ℒ\left(D\;|\;L,\tau_{1},\ldots ,\tau_{L};{\bar{S}}_{1},\ldots ,{\bar{S}}_{L}\right)=\prod_{i=1}^{N}\left[\sum_{j=1}^{L}\tau_{j}ℱ\left({\bar{f}}_{i}\;|\;{\bar{S}}_{j}\right)\right].$$

To infer the true sequences and their frequencies we maximise this likelihood using an expectation-maximization (EM) algorithm. EM algorithms apply very naturally to complex clustering problems. Intuitively they exploit the fact that if the properties of the cluster centres are known, then the probability that a given data point was generated by a given cluster centre is easy to calculate, similarly if those probabilities are known then maximum likelihood solutions for the parameters of the cluster centres can be calculated. What is hard to do are these two steps simultaneously. In an EM algorithm we avoid that by iterating the two steps separately until the parameters converge at a local maximum of the likelihood [16]. The algorithm used here differed in two ways from that proposed previously [5]. Firstly, we did not align flowgrams to our denoised sequences before calculating the distances. That was necessary to allow for PCR errors that cause changes resulting in insertions and deletions at the flowgram level. Flowgram gaps do occur very rarely as a result of pyrosequencing noise, occasionally no signal ≥ 0.5 is observed in a frame of four nucleotides, but as described above we truncated our flowgrams when this was observed. Consequently by not performing alignments we ensured that only pyrosequencing noise would be filtered at this step. Secondly, we did not construct the maximum likelihood sequences each flow at a time, instead we only allowed sequences that were observed in the data. This allows the final denoised reads to be mapped to the originals.

We will now explain the EM algorithm in detail. We represent the mapping of data points to clusters, or in our case flowgrams to sequences, by a matrix *Z *where each row *i *corresponds to a flowgram and contains only zeros and a single one at the column *j *indexing the sequence that generated it. This can be expressed using Kronecker deltas *z_i, j _*= *δ*_*i, m*(*i*) _where *m*(*i*) gives the sequence that generated flowgram *i*. We now define a complete data likelihood that includes both the observed data and this matrix of unobserved mappings:

(3) $$ℒC\left(D,Z\;|\;L;\tau_{1},\ldots \tau_{L};{\bar{S}}_{1},\ldots ,{\bar{S}}_{L}\right)=\prod_{i=1}^{N}\prod_{j=1}^{L}{\left[\tau_{j}ℱ\left({\bar{f}}_{i}\;|\;{\bar{S}}_{j}\right)\right]}^{z_{i,j}},$$

assuming that each row of *Z*, the vector ${\bar{z}}_{i}$, is i.i.d according to a multinomial over *L *categories with probabilities *τ*_1_,...,*τ_L_*. We then define the quantity ${\hat{z}}_{i,j}$ as the conditional expectation of *z_i, j _*given the model parameters, i.e. the sequences and their abundances, under the complete data likelihood. These are the conditional probabilities that sequence *j *generated flowgram *i*. The EM algorithm iterates between an E step, where the ${\hat{z}}_{i,j}$ are computed given the model parameters and an M step, where the model parameters are calculated so as to maximize Equation 3 with the *z_i, j _*replaced by their estimates ${\hat{z}}_{i,j}$. This process will, under quite general conditions, converge to a local maximum of Equation 2 [16]. Our EM algorithm is:

1. M step: Given the ${\hat{z}}_{i,j}$ set each sequence ${\bar{S}}_{j}$ to the sequence corresponding to the perfect flowgram *Ū_j _*that maximises Equation 3, restricted to the set of *P *unique perfect flowgrams ${\bar{Q}}_{k}$ obtained by rounding the observed flowgrams to integers. This corresponds to finding the perfect flowgram with the smallest total distance to all the reads weighted by the conditional probabilities that each flowgram was generated by that denoised sequence:

(4) $${\bar{U}}_{j}={\bar{Q}}_{l}\text{ where }l=\underset{k}{\min}\left[\sum_{i=1}^{N}{\hat{z}}_{i,j}d^{\prime }\left({\bar{f}}_{i},{\bar{Q}}_{k}\right)\right]$$

Define new relative frequencies as $\tau_{j}=\sum_{i=1}^{N}{\hat{z}}_{i,j}\;/N$. This generates sequences and their frequencies which maximize the complete data likelihood of Equation 3 given *D *and $\hat{Z}$.

2. Calculate new distances $d^{\prime }({\bar{f}}_{i},{\bar{U}}_{j})$.

3. E step: Calculate new ${\hat{z}}_{i,j}$ as:

(5) $${\hat{z}}_{i,j}=\frac{\tau_{j}\exp\left(-\frac{d^{\prime }({\bar{f}}_{i},{\bar{U}}_{j})}{\sigma_{p}}\right)}{\sum_{k=1}^{L}\tau_{k}\exp\left(-\frac{d^{\prime }({\bar{f}}_{i},{\bar{U}}_{k})}{\sigma_{p}}\right)}.$$

4. Repeat until convergence

Expectation-maximization algorithms because they only find local optimum are sensitive to initial conditions. To initialise the EM algorithm we performed a complete linkage hierarchical clustering based on flowgram distances and formed clusters at a given cut-off, *c_p_*. This also defines the number of denoised sequences *L*, although the number with non-zero weight *τ_j _*usually decreases during the iteration. The pyrosequencing noise removal therefore has two parameters *σ_p _*and *c_p_*, for all the results presented here these were set at the values 1/60 and 0.01 respectively.

### Removing PCR Noise

The advantage of splitting the pyrosequencing and PCR noise removal steps is that it allows a more appropriate model to be used for the removal of the PCR single base errors. We used the same ideas as above to develop a procedure for removing PCR errors. We define a distance that reflects the probability that a given read $\bar{r}$ could have been generated from a true sequence $\bar{S}$, given PCR error. This probability is simply the sum of the necessary nucleotide transitions i.e. the probability that a nucleotide *m *is observed when the true nucleotide is *n*, *P *(*m*|*n*). The total probability of the read will be the product of these and we take the negative logarithm to generate a sequence error corrected distance between zero and infinity. We also normalise by the alignment length *A*:

(6) $$\begin{array}{c} e\left(\bar{r},\bar{S}\right)=-log\left[P\left(\bar{r}\;|\;\bar{S}\right)\right]\\ =-log\left[\prod_{l=1}^{M}P\left(r^{l}=m\;|\;s^{l}=n\right)\right]/A\\ =\sum_{l=1}^{A}-log\left[P\left(r^{l}=m\;|\;s^{l}=n\right)\right]/A \end{array}$$

This requires alignment of the read to the sequence. Alignment was performed with a specially modified version of the Needleman-Wunsch algorithm with a reduced gap cost for homopolymer insertion and deletions. This accounted for the possibility of pyrosequencing noise on low frequency reads which may not have been removed in the flowgram clustering. Gap penalties were included in the distance measure.

The nucleotide transition probabilities were calculated by comparing all reads with pyrosequencing noise removed from the three 'Even' V2 data sets with the known control sequences. These are shown in Table 2. It is interesting to note the higher frequencies of transitions (*A *↔ *G *and *C *↔ *T *) versus transversions. This was also found for the V5 and Titanium data. Indeed the relative magnitudes of these probabilities were similar for all the data sets, perhaps because standard Taq polymerases were used throughout, and the per cycle error rates were the same order of magnitude as has been observed by other methods for Taq polymerases [17]. We therefore used the transition probabilities in Table 2 for calculating sequence distances in all the data sets.

**Table 2:** PCR per base error probabilities for the three 'Even' V2 'Mock Communities'

Nucleotide	A	C	T	G	Total *f*	Per cycle *p*
A	0.9995	7.2e-6	7.7e-6	5.1e-4	5.2e-4	3.5e-5
C	1.1e-05	0.9996	4.1e-4	2.1e-6	4.2e-4	2.8e-5
T	9.0e-6	5.7e-4	0.9994	1.4e-5	5.9e-4	4.0e-5
G	3.5e-4	3.2e-6	2.1e-5	0.9996	3.7e-4	2.5e-5

The rows give the true nucleotides and the columns the observed as a result of errors. Estimates of the individual probabilities are given as frequencies. The total error probability *f *for each nucleotide is also quoted in the penultimate column, followed by the per cycle error rate *p *calculated as *p *= 2*f */*n*, where *n *= 30 is the cycle number [8,17].

We used a mixture model to cluster the sequences, just as for pyrosequencing noise removal, where each component of the mixture corresponds to a true sequence about which observed noisy reads are distributed. The relative weights of each component are the true relative frequencies of the sequences. The reads are assumed to be distributed as exponentially decaying functions of their sequence error corrected distance from these true sequences. The magnitude of the sequence noise is described by the characteristic length of these exponentials, *σ_s _*. A maximum likelihood fit of the mixture model can be obtained using an Expectation-Maximization algorithm initialised using the clusters formed from a hierarchical clustering of sequences at a given distance cut-off, *c_s _*. In this study we used parameter values of *σ_s _*= 0.033 and *c_s _*= 0.08, parameters that experience has taught us work well for GS FLX data. For the Titanium data we compared two different values for *σ_s_*, 0.1 and 0.04, whilst keeping *c_s _*= 0.08. A standard gap was given a penalty of 15.0 and a homopolymer gap, 4.0. Prior to our sequence clustering step we truncated at 220 bp for GS FLX and 400 bp for Titanium because of the increase in error rates at the ends of reads.

### Chimera Identification

Chimeras are generated when incomplete extension occurs in one round of PCR and then the resulting sequence fragment acts as a primer for a different sequence in the next round. Consequently chimeras are composed of two (or occasionally more) true sequences with a discrete break point where the transition from one sequence to another occurs. For our nine test data sets we were therefore able to determine which sequences after denoising were likely chimeras by aligning each sequence against the known reference sequences and finding the putative parents and break point which gave the closest match to the query sequence. If the closest match to a chimera of two sequences was at least three nucleotides or better than that to a single reference sequence then the query was considered as a possible two sequence chimera or 'Bimera'. If it was not then it was considered a 'Good' sequence. Similarly if the match was further improved by three nucleotides when two break points were allowed then it was classified as possibly comprised of three sequences a 'Trimera', and again for the transition to a composite of four sequences or 'Quadramera'. However, the sequence was only classified to these putative definitions if the absolute match was sufficiently good as measured in terms of the sequence error corrected distance (*e *< 0.15 a distance corresponding to one non-homopolymer gap per 100 nucleotides). Otherwise the sequence was denoted as 'unclassified'. These could include contaminants, real unidentified 16S rRNA operons, gross pyrosequencing or PCR errors, or most likely a chimera that failed to fall under our rather strict definition.

### Perseus: Chimera Removal as a Problem in 'Supervised Learning'

For real pyrosequencing data we will not know *a priori *what sequences should be present and therefore chimera identification algorithms are necessary. Given the mechanics of PCR amplification, any chimeras generated will experience at least one less PCR cycle than either parent, consequently both parents of a chimera will be present in the data set and with a frequency at least equal to the chimera. This ignores the possibility of the chimera experiencing preferential PCR bias over its parents, but it will be true in the vast majority of cases. To exploit this observation we developed an algorithm 'Perseus' that considers each sequence in turn and performs exact pairwise alignments to all sequences with equal or greater abundance, the set of possible parents. The two parents and break point that give the closest match to the query are then identified and a three way alignment of these sequences is generated using the mafft-linsi program [18]. We calculate two quantities from this alignment - the first is the PCR error corrected distance from the query to the optimum chimera. For a sequence to be classified as chimeric this distance has to be absolutely smaller than 0.15 and smaller than or equal to the distance to the closest sequence amongst the best possible parents. This simply ensures that the hypothesis that the sequence is a chimera is possible. However, we still have to account for the possibility that the chimeric pattern could have evolved. We do this by calculating a second quantity using the alignment the 'chimera index' *I *.

Denoting the query sequence $\bar{C}$, the closest matching parent $\bar{A}$, and the more distant parent $\bar{B}$, we calculate using parsimony the sequence ancestral to all three. We find the number of base pair changes along the three branches to $\bar{A}$, $\bar{B}$, $\bar{C}$ and denote these *x*, *y*, and *z *respectively. We resolve changes to the two parts of alignment, either the part of the chimera matching parent $\bar{A}$ or parent $\bar{B}$, and denote these *x_A _*and *x_B _*, *y_A _*and *y_B _*, and *z_A _*and *z_B _*. For a given chimera to be observed, two independent events must occur, changes to the distant parent $\bar{B}$ must occur on that part of the alignment matching $\bar{A}$. Assuming all base changes are equally likely then the distribution of changes across the two parts will be binomially distributed with probability proportional to the size of each part. Therefore, we can calculate the probability of the changes being as biased or more so than were observed. The same arguments apply for the changes to the closer parent, they should all lie in the part matching the more distant and we can calculate that probability. We then multiply the two probabilities together and take the negative log to obtain an index that will increase the less likely a chimeric pattern is to have evolved. This index is defined for two parent chimeras, our so called 'bimeras', it could be extended to higher order chimeras but we did not do this, finding that it sufficed for identifying these anyway.

Having defined this index the problem of identifying chimeras becomes an example of supervised learning with our labeled test data sets as training and validation data. We used the test data sets with equal abundances, the V5 'Divergent' data set, and the three V2 'Even' mock communities for training. We calculated *I *for each sequence that satisfied the chimera matching criteria and then used the known classifications to either good or chimeric ('Bimera'), determined through comparison with the reference sequences to train a one dimensional logistic regression on *I *separately for the V2 and V5 sequences [19]. This procedure allows us to account for our assumption that all bases are equally likely to evolve. A logistic regression assumes that the probability of a sequence being chimeric can be expressed as:

(7) $$P\left(\bar{S}=C\right)=\frac{1}{1+exp\left(-\left[\alpha +\beta I\right]\right)}.$$

We also added to our training data set the result of taking the reference and calculating their indices without regard to sequence frequency: i.e., comparing all sequences to all others. We then used the test data sets with uneven abundances, the V5 'Artificial Community' and the three V2 'Uneven' data sets, for validation. Running them through our algorithm and then using the logistic regressions to generate probabilities of each sequence being chimeric. Those sequences that do not have a good chimeric match have this probability set to zero. We then defined all sequences with a probability of greater than 50% of being chimeric as chimeras. This will minimise total misclassifications [19]. We also trained the classifier with the Titanium V4-V5 data and associated reference sequences but in this case we lacked a separate data set for validation.

## Results and Discussion

In addition to running the AmpliconNoise pipeline we also denoised the data sets with the DeNoiser algorithm [9] and using single-linkage preclustering (SLP) at the recommended 2% sequence difference as well as at 1% for comparative purposes [10]. We truncated the reads at 220 bp and 400 bp for GS FLX and Titanium respectively before calculating exact pairwise sequence distances for the SLP algorithm. For SLP we used the same filtered reads as for AmpliconNoise but this was not possible for the DeNoiser since there filtering is through the QIIME pipeline [20]. The QIIME filtering is slightly less stringent than the procedure described above for GS FLX data but more so for Titanium where a quality window is recommended. The read numbers following QIIME filtering are also given in Table 1.

### Per base error rates following noise removal

The first comparison we will make between the different noise removal algorithms is to calculate average per base error rates. To determine if the algorithms really do reduce noise we will also compare to the raw reads. To calculate error rates in the raw reads we simply compared each read to its closest match amongst the reference sequences, aligned, calculated the number of differences, and the alignment length. The per base error rate was then estimated as the sum of the differences divided by the sum of the lengths for the whole data set. The results are given in the first column of Table 3. The raw error rates vary slightly across data sets, the V5 - GS FLX and V4-V5 error rates are similar at around 0.4% slightly higher than the 0.25% reported previously [4] but this is substantially increased to more than twice that value in the V2 data sets.

**Table 3:** Percentage per base error rates in pyrosequencing reads before and after application of denoising algorithms

Name	Raw	AmpliconNoise	SLP 1%	SLP 2%	DeNoiser
Divergent	0.4519%	0.1877%	0.8030%	0.7960%	0.1987%
Artificial	0.3678%	0.2469%	1.1733%	1.1891%	0.5605%
Even1	0.8767%	0.6381%	1.6990%	2.3572%	0.7585%
Even2	0.8870%	0.6456%	1.7906%	2.1536%	0.7986%
Even3	0.8446%	0.5935%	1.9895%	2.4594%	0.8033%
Uneven1	1.1852%	0.8998%	1.9687%	1.7756%	1.0897%
Uneven2	0.6713%	0.4851%	1.7549%	2.6078%	0.6646%
Uneven3	0.6560%	0.4344%	1.3370%	2.0822%	0.4913%
Titanium - *σ_s _*= 0.1	0.4576%	0.1427%	0.5830%	0.7012%	0.3793%
Titanium - *σ_s _*= 0.04		0.1325%			

Noise removal by all the algorithms can be considered a form of mapping. We map a noisy read onto another that we believe really generated that read. To calculate per base error rates after noise removal we must account for the possibility that the mapping may be to the wrong read. To allow for this we estimated the denoised error rates by, for each read, calculating the number of differences between the denoised read it maps to and the closest matching reference of the original undenoised read. The total of the differences across the data set was then normalised by total alignment length to estimate the per base error rate. The results for the four algorithms are given in Table 3. For the DeNoiser results we only used those reads that were included in the AmpliconNoise and SLP data sets ensuring a fair comparison despite the slight differences in filtering. What is immediately apparent is that SLP at both cut-offs does not actually remove errors instead it inflates them. This is due to the high rate of misassignment where a read is mapped not onto the reference that generated it but to a similar but incorrect sequence. The DeNoiser algorithm does better reducing per base error rates in most cases but it is substantially out-performed by AmpliconNoise which is capable of reducing noise by one-third to a half in all data sets. Given that some residual error will always remain because the sequencing of the references may not be entirely accurate and because of PCR chimeras and contaminants then this is impressive.

### Relative Importance of Pyrosequencing and PCR noise

**Figure 2:**
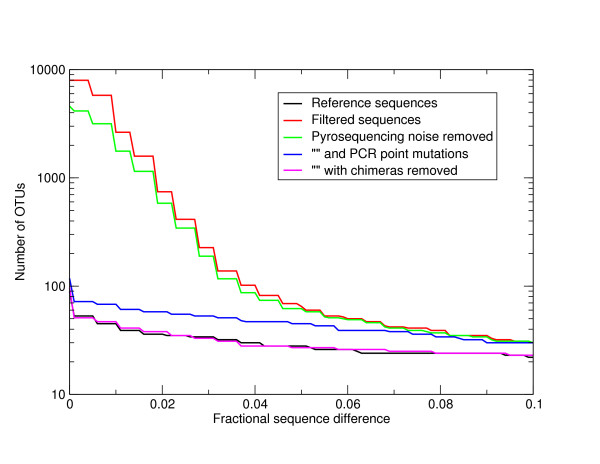
**OTU numbers in the V5 'Artificial Community' as a function of percent sequence difference - logarithmic**. Numbers of OTUs formed at cut-offs of increasing percent sequence difference after complete linkage clustering of the 'Artificial Community' V5 data set (Table 1). Distances were calculated following pair-wise alignment with the Needleman-Wunsch algorithm. Results are shown following filtering (red line), pyrosequencing noise removal by the first PyroNoise stage of AmpliconNoise (green line), further removal of PCR point mutations by the second SeqNoise stage (blue line) and following removal of chimeric sequences (magenta line). For comparison the number of OTUs obtained by clustering the known reference sequences are shown in black. The y-axis is logarithmically scaled.

The effect of these errors will be to inflate estimates of OTU number. In order to quantify the relative importance of pyrosequencing and PCR noise to the excess of OTUs observed at different levels of sequence difference we calculated OTU number following complete linkage clustering as a function of percent sequence difference for the 'Artificial Community' data set. We used the exact pairwise Needleman-Wunsch algorithm to calculate distances between sequences prior to OTU formation. This removed the potential complicating factor of incorrect multiple alignments [21]. We used a complete linkage hierarchical clustering and took clusters at increments of 0.1% nucleotide difference to form OTUs. Complete linkage is more sensitive to noise than the alternative average linkage algorithm [5], but it gives OTUs with a closer correspondence to taxonomic classifications [22]. The OTU numbers are shown in Figure 2. This graph is logarithmically scaled. We generated OTUs for the filtered sequences (red line), this gives an indication of the total effect of errors, the sequences after pyrosequencing noise removal by the first PyroNoise stage of AmpliconNoise (green line), and following removal of PCR point mutations by the second SeqNoise stage (blue line). The clustering of the reference sequences is shown by the black line, this indicates the true OTU diversity in the sample. Pyrosequencing errors account for roughly half of the extra diversity (note the logged scale), the majority of the rest derive from PCR point mutations. However, even after applying both noise removal steps there is still an excess of OTUs, these we hypothesized were due to the formation of PCR chimeras.

### Chimera Frequencies

For each of the data sets following noise removal by the different algorithms, the number of denoised sequences classified as either 'Good', one of our three classes of chimeric sequences 'Bimera', 'Trimera', 'Quadramera', or 'Unclassified' are given in Table 4. Just focusing on the AmpliconNoise denoised samples then these results illustrate the potential importance of chimeric reads in pyrosequenced amplicons. What is most striking is the difference between the V5 and V2 sequences. In the former the total number of unique sequences classified as chimeric was about 25%, with slightly more chimeric sequences in the 'Divergent' as opposed to the 'Artificial Community'. For the latter it was still true that the data sets with evenly mixed references had a higher proportion of chimeric sequences but the number of chimeric sequences was far higher. In fact only some 5-10% of sequences could be mapped back to the reference sequences. This provides ideal data sets for training and testing chimera identification algorithms. Similar patterns were seen for the other algorithms although exact numbers and frequencies vary. The DeNoiser algorithm achieves a higher frequency of good sequences on the V2 mock communities but as we discuss below this is due to over-clustering.

**Table 4:** The classification of denoised sequences from the nine test data sets

Name	Unique	Good	Bimera	Trimera	Quadramera	Unclassified
Divergent						

AmpliconNoise	79	56(70.9%)	21(26.6%)	1(1.3%)	0 (0.0%)	1(1.3%)
SLP 1%	305	210 (68.9%)	23 (7.5%)	1 (0.3%)	0 (0.0%)	71(23.3%)
SLP 2%	60	29 (48.3%)	22 (36.7%)	0 (0.0%)	0 (0.0%)	9(15.0%)
DeNoiser	37	28 (75.7%)	7 (18.9%)	1 (2.7%)	0 (0.0)	1(2.7%)

Artificial						

AmpliconNoise	118	94(79.7%)	21(17.8%)	0 (0.0%)	0 (0.0%)	3(2.5%)
SLP 1%	230	168 (73.0%)	21 (9.1%)	0 (0.0%)	0 (0.0%)	41(17.9%)
SLP 2%	62	36 (58.1%)	20 (32.3%)	0 (0.0%)	0 (0.0%)	6(9.7%)
DeNoiser	59	46 (78.0%)	8 (13.6%)	0 (0.0%)	0 (0.0%)	5(8.5%)

Even1						

AmpliconNoise	2341	89 (3.8%)	1847 (78.9%)	244 (10.4%)	4 (0.2%)	157(6.7%)
SLP 1%	2205	108 (4.9%)	1631 (74.0%)	253 (11.5%)	4 (0.2%)	209(9.5%)
SLP 2%	894	38 (4.3%)	638 (71.4%)	142 (15.9%)	4 (0.4%)	72(8.1%)
DeNoiser	289	63 (21.8%)	161 (55.7%)	36 (12.5%)	3 (1.0%)	26(9.0%)

Even2						

AmpliconNoise	2082	90 (4.3%)	1651 (79.3%)	227 (10.9%)	4 (0.2%)	110(5.3%)
SLP 1%	1958	97 (5.0%)	1448 (74.0%)	235 (12.0%)	4 (0.2%)	174(8.9%)
SLP 2%	789	44 (5.6%)	553 (70.1%)	134 (17.0%)	4 (0.5%)	54(6.8%)
DeNoiser	285	64 (22.5%)	171 (60.0%)	22 (7.7%)	4 (1.4%)	24(8.4%)

Even3						

AmpliconNoise	2210	91 (4.1%)	1781 (80.6%)	188 (8.5%)	3 (0.1%)	147(6.7%)
SLP 1%	2164	117 (5.4%)	1635 (75.6%)	194 (9.0%)	3 (0.1%)	215(9.9%)
SLP 2%	874	40 (4.6%)	649 (74.3%)	105 (12.0%)	1 (0.1%)	79(9.0%)
Denoiser	287	64 (22.3%)	170 (59.2%)	24 (8.4%)	0 (0.0%)	29(10.1%)

Uneven1						

AmpliconNoise	1124	94 (8.4%)	816 (72.6%)	81 (7.2%)	1 (0.1%)	132(11.7%)
SLP 1%	1040	90 (8.7%)	682 (65.6%)	88 (8.5%)	2 (0.2%)	178(17.1%)
SLP 2%	439	51 (11.6%)	278 (63.3%)	49 (11.2%)	1 (0.2%)	60(13.7%)
Denoiser	212	61 (28.8%)	111 (52.4%)	9 (4.2%)	0 (0.0%)	31(14.6%)

Uneven2						

AmpliconNoise	859	77 (9.0%)	669 (77.9%)	71 (8.3%)	2 (0.2%)	40(4.7%)
SLP 1%	814	81 (10.0%)	570 (70.0%)	77 (9.5%)	2 (0.2%)	84(10.3%)
SLP 2%	330	36 (10.9%)	226 (68.5%)	36 (10.9%)	2 (0.6%)	30(9.1%)
Denoiser	154	49 (31.8%)	87 (56.5%)	9 (5.8%)	1 (0.6%)	8(5.2%)

Uneven3						

AmpliconNoise	1053	75 (7.1%)	843 (80.1%)	82 (7.8%)	0 (0.0%)	53(5.0%)
SLP 1%	1031	89 (8.6%)	745 (72.3%)	92 (8.9%)	0 (0.0%)	105(10.2%)
SLP 2%	399	45 (11.3%)	259 (64.9%)	49 (12.3%)	0 (0.0%)	46(11.5%)
Denoiser	202	55 (27.2%)	124 (61.4%)	7 (3.5%)	0 (0.0%)	16(7.9%)

Titanium						

AmpliconNoise - *σ_s _*= 0.1	163	76 (46.6%)	77 (47.2%)	1 (0.6%)	0 (0.0%)	9(5.5%)
AmpliconNoise - *σ_s _*= 0.04	304	91 (29.9%)	174 (57.2%)	2 (0.7%)	0 (0.0%)	37(12.2%)
SLP 1%	765	520 (68.0%)	157 (20.5%)	1 (0.1%)	0 (0.0%)	87(11.4%)
SLP 2%	182	72 (39.6%)	92 (50.5%)	1(0.5%)	0 (0.0%)	17(9.3%)
DeNoiser	151	14 (9.3%)	70 (46.4%)	6(4.0%)	0 (0.0%)	61(40.4%)

The number of unique denoised sequences classified into the five categories, 'Good', 'Bimera', 'Trimera', 'Quadramera' and 'Unclassified', as defined in the text are given together with percentages of the total in parentheses.

### Accuracy of OTU Construction Following Noise Removal

**Figure 3:**
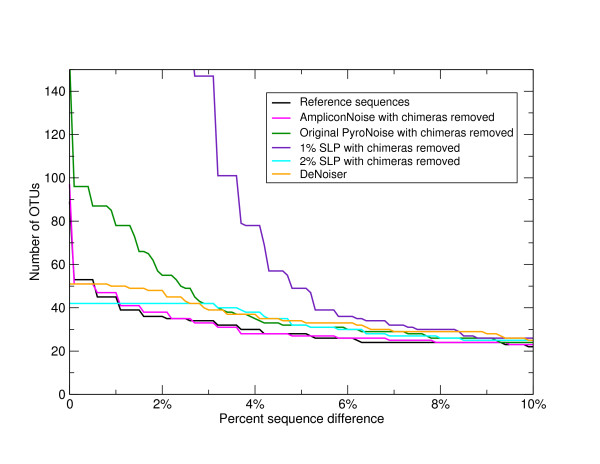
**OTU numbers in the V5 'Artificial Community' as a function of percent sequence difference - linear**. Numbers of OTUs formed at cut-offs of increasing percent sequence difference after complete linkage clustering of the 'Artificial Community' V5 data set (Table 1). Distances were calculated following pair-wise alignment with the Needleman-Wunsch algorithm, results are shown for the filtered sequences after pyrosequencing and PCR noise removal by AmpliconNoise (magenta line), for single-linkage preclustering at 1% (purple) and SLP at 2% (cyan), for the DeNoiser algorithm (orange), and for the original one-stage PyroNoise algorithm (dark green line). In all cases chimeric sequences were removed. For comparison the number of OTUs obtained by clustering the known reference sequences are shown in black. The y-axis is scaled linearly.

Following noise removal with AmpliconNoise, we removed those sequences classified as chimeric from the 'Artificial Community' data and rebuilt OTUs. The results are shown as a magenta line in Figure 2. It can be seen that the OTU diversity now closely tracks that of the reference sequences. This illustrates that assuming we can remove chimeras then we are getting the right number of OTUs following noise removal above a sequence difference of just over 1%. This is a significant improvement over the original PyroNoise program as can be seen from Figure 3 where we repeat the AmpliconNoise results together with the OTU numbers from our original one-stage clustering but with a linearly scaled y-axis. AmpliconNoise is also considerably faster than the original PyroNoise. In Table 5 we give the time in seconds taken to run the 'Artificial Community' data using 64 cores of a Linux cluster for the two algorithms. The times are resolved across the individual steps. This includes an initial calculation of pairwise sequence distances and an average linkage clustering which is used to split up the data set which is common to both. Both wall times i.e. time from start to end of the program and the CPU times actually spent on calculations are given. The latter is summed over all cores and is therefore considerably larger. The total wall time for AmpliconNoise is around seventy seven minutes compared to over ten times longer for the original PyroNoise.

**Table 5:** Run times in seconds for AmpliconNoise and the original PyroNoise for the V5 'Artificial Community' data set

	AmpliconNoise			PyroNoise		
**Process**	**Cores**	**Wall**	**CPU**	**Cores**	**Wall**	**CPU**

Initial pairwise distances (NDist)	64	1900	45358	64	1900	45358
Initial clustering (FCluster)	1	292	252	1	292	252
Flowgram clustering (PyroNoise)	64	1904	9364	64	55241	686497
Sequence clustering (SeqNoise)	64	503	20713	--	--	--

Total		4599	75687		57433	732107

The results are for a Xeon Linux cluster running programs across a maximum of 64 cores. Two times in seconds are quoted for each step, wall (time) is the true time elapsed between the start and end of a process and CPU (time) is the total time over all cores spent on actual calculations.

In Figure 3 we also show the effect of applying single-linkage preclustering at 1% and the recommended 2% level, and the DeNoiser algorithm prior to OTU construction. Single-linkage preclustering at 1% is very poor greatly over-estimating OTU numbers, 2% SLP and the DeNoiser perform similarly above a 3% cut-off predicting slightly more OTUs than the original PyroNoise program, both were worse than AmpliconNoise. Below this 2% SLP predicts a constant OTU number suggesting it is aggregating OTUs that should be separated. However, both SLP and DeNoiser were orders of magnitude faster than AmpliconNoise, single-linkage preclustering does not require a cluster and for this data set it ran in just a few minutes on a standard computer. The DeNoiser took less than an hour on a single core.

**Figure 4:**
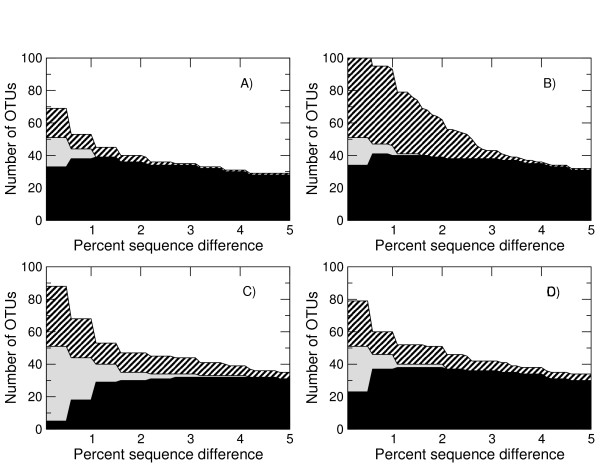
**OTU construction accuracy for the V5 'Artificial Community' as a function of percent sequence difference for the different noise removal algorithms**. Results are given for the improved two stage 'AmpliconNoise' (A), the original 'PyroNoise' (B), single-linkage preclustering at 2% (C), and the DeNoiser algorithm (D). Reads classified as chimeric by comparison with the references were removed. The solid black portion gives the number of OTUs comprised of reference sequences and denoised pyrosequenced reads. These are good OTUs. The grey area OTUs formed only from reference sequences. These correspond to true OTUs that are missed. The diagonal shaded area those OTUs containing only pyrosequenced reads and hence are noise.

To investigate whether we are not only getting the right numbers of OTUs but also the right OTUs, we built OTUs using both the V5 reference sequences and the denoised sequences following removal of those classified as chimeras. Having done this those OTUs that contain both reference and denoised sequences indicate true diversity that we have observed we classified these as 'Good', those OTUs that only contain denoised sequences can be considered 'Noise', and those OTUs only containing reference sequences indicate diversity that we have 'Missed'. The numbers of each as a function of percent sequence difference for AmpliconNoise applied to the 'Artificial Community' following noise removal are shown in Figure 4A. This indicates that above about 1.5% we are getting all the OTUs that should be there with only one or two noisy OTUs. There is a substantial improvement over the original one-step version of PyroNoise in terms of reduction in OTUs attributable to noise (Figure 4B) and a very slight improvement in terms of capturing the diversity that is there. It is also substantially better than the performance of 2% single-linkage preclustering (Figure 4C). Using SLP noise reduction is not nearly as good, at 3% there are 10 OTUs attributable to noise as opposed to 5 for the original PyroNoise and just one for AmpliconNoise. More disturbingly, we now fail to capture all the OTUs that should be present, this is obviously to be expected at cut-offs below 2% but we still miss two OTUs at the 3% level. For this data set the DeNoiser does better than 2% SLP getting all diversity above 2% cut-off with noise comparable to the original PyroNoise.

In order to determine if these results generalise we built 3% OTUs for each set of denoised sequences together with the corresponding reference sequences for all the data sets. We then classified the 3% OTUs as above. The results are given in Table 6. From this table we see that AmpliconNoise is the only algorithm that consistently obtains all the true diversity at 3%. Only in the V2 data set 'Uneven3' is a single OTU missed. For the V5 and Titanium data sets it simultaneously removes a large proportion of the noise. For the V5 data a significant number of noisy OTUs remain. However, this reflects the unusual frequency of chimeras in this data, a large proportion of those noisy OTUs likely being chimeras that differed by only one or two nucleotides from their closest parent and consequently not classified as chimeric under the rather strict definition given above. The two agglomerative algorithms perform substantially worse. Across all the data sets SLP at 1% is incapable of reducing noise to reasonable levels, whilst SLP at 2% has missing 3% OTUs in all the data sets, and for the V2 data does extremely poorly in this respect, at the same time it fails to reduce the number of noisy OTUs below that of AmpliconNoise for the higher quality V5 and Titanium data sets. The DeNoiser performs adequately for the V5 data and for the V2 GS FLX 'Mock Communities' the number of noisy OTUs is actually lower for the DeNoiser than AmpliconNoise but just as for 2% SLP this comes at a cost of a substantial number of missed OTUs, around 10% of the true diversity. A situation that gets dramatically worse for the Titanium data set where over 75% of OTUs are missing. It is likely that this is due to high frequency chimeras being identified as cluster centers and then good sequences of lower frequency are being mapped onto them.

**Table 6:** Accuracy of 3% OTU construction following application of the noise removal algorithms

Name	AmpliconNoise			SLP 1%			SLP 2%			DeNoiser		
**3% OTUs**	**Good**	**Missed**	**Noise**	**Good**	**Missed**	**Noise**	**Good**	**Missed**	**Noise**	**Good**	**Missed**	**Noise**

Divergent	26	0	1	26	1	162	26	1	11	24	0	3
Artificial	34	0	1	38	0	109	32	2	10	36	0	6
Even1	57	0	131	60	2	174	39	19	65	59	1	21
Even2	57	0	89	59	2	137	42	15	53	58	3	22
Even3	58	0	125	65	0	197	43	17	75	58	3	23
Uneven1	58	0	101	60	0	144	47	10	59	54	7	24
Uneven2	48	0	35	51	0	74	38	10	26	46	6	6
Uneven3	49	1	46	51	2	97	41	10	45	48	6	12

Titanium												

*σ_s _*= 0.1	66	0	8	74	0	169	67	1	18	15	53	14
*σ_s _*= 0.04	67	0	21									

Reads classified as chimeric by comparison with the references were removed prior to complete linkage OTU construction at 3% sequence difference with the corresponding reference sequences. Each OTU was then classified as 'Good' if it comprised reference sequences and denoised pyrosequenced reads, 'Missed' OTUs are formed only from reference sequences, and those OTUs containing only pyrosequenced reads are designated 'Noise'. The numbers of classified OTUs observed for each data set and noise removal algorithm are shown below.

**Figure 5:**
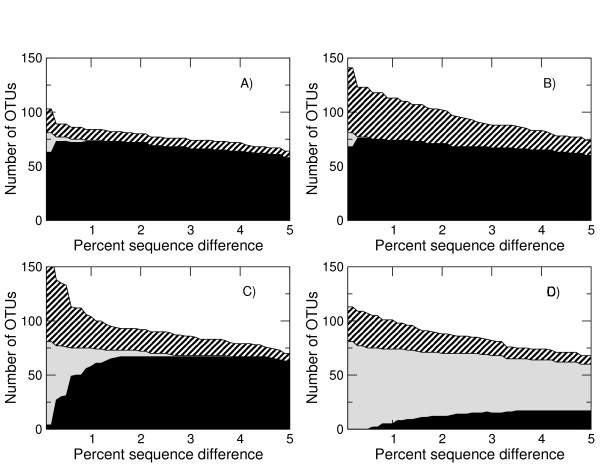
**OTU construction accuracy for the Titanium data set as a function of percent sequence difference for the different noise removal algorithms**. Results are given for AmpliconNoise with *σ_s _*= 0.01 (A) and *σ_s _*= 0.04 (B), single-linkage preclustering at 2% (C), and the DeNoiser algorithm (D). Reads classified as chimeric by comparison with the references were removed. The solid black portion gives the number of OTUs comprised of reference sequences and denoised pyrosequenced reads. These are good OTUs. The grey area OTUs formed only from reference sequences. These correspond to true OTUs that are missed. The diagonal shaded area those OTUs containing only pyrosequenced reads and hence are noise.

We investigate the Titanium data more fully in Figure 5, where as in Figure 4, we show the number of good, missing and noisy OTUs as a function of per cent sequence cut-off. This shows that AmpliconNoise with *σ_s _*= 0.01 (Figure 5A) is capable of removing most noise and retaining all diversity above about 1%, with the smaller cluster size *σ_s _*= 0.004 it is possible to obtain all the OTUs that should be present down to just 0.5% but at the cost of increased noise (Figure 5B). Single-linkage preclustering at 2% once again fails to get all the OTUs that should be there even at 3% (Figure 5C) and the DeNoiser does very badly missing a large portion of the true diversity (Figure 5D).

### Chimera Classification Accuracy

**Figure 6:**
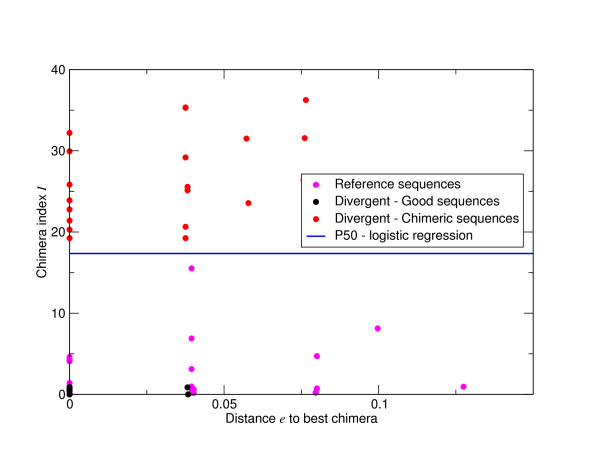
**Training logistic regression on denoised V5 'Divergent' data**. Good sequences are shown as black dots, chimeras red and reference sequences magenta. We used the denoised V5 'Divergent' data set, classified either good or chimeric by comparison with the references, and the reference sequences, all good, to train a one dimensional logistic regression on the 'chimera index' *I *using the R software package [30]. An intercept, *α *= - 183.25, and coefficient, *β *= 10.56, were obtained despite the fact that the algorithm did not converge (see text), and the corresponding P50 classification value, 17.35, is shown (blue line).

**Figure 7:**
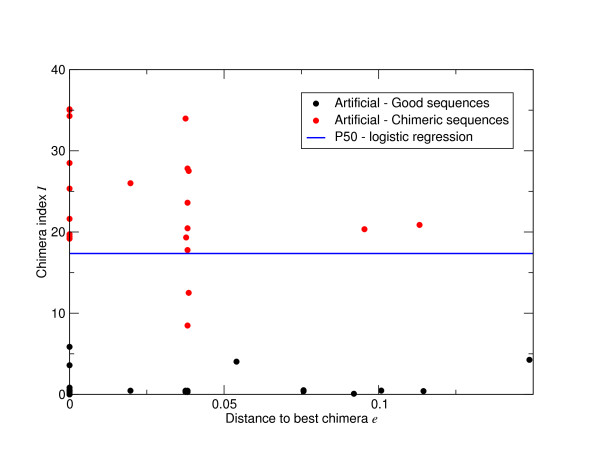
**Validation of logistic regression on denoised V5 'Artificial Community' data**. Applying the classification rule (blue line) from Figure 5 to the 'Artificial Community' denoised data sets correctly predicts all but two chimeras that fall below the P50 line. Good sequences are shown as blackdots and chimeras red.

The results of the training on the V5 'Divergent' denoised sequences from AmpliconNoise are shown in Figure 6. In this case the logistic regression on *I *can exactly separate the good and reference sequences from the chimeras. A consequence of this is that the algorithm fails to converge predicting a decision line, a P50 value corresponding to a 50% probability of a sequence being chimeric, but being unable to predict uncertainty about that line essentially because there is none. The results of applying this classification rule to the denoised V5 'Artificial Community' are shown in Figure 7. Two chimeras fall below the P50 line and hence with 50% probability cut-off would be falsely classified as good. There were no false positives. When we applied ChimeraSlayer to these sequences we missed 13/20 chimeras at the suggested 90% bootstrap, at 50% which perhaps better reflects our classification rule it misses 7/20 but at the cost of 11/94 false positives. We also performed a further logistic regression on all the data, both the 'Divergent' and 'Artificial Community' data sets and the reference sequences. This highly significant fit (Null deviance 208.819 on 206 degrees of freedom, residual deviance 11.007 on 205 degrees of freedom, AIC: 15.007) gave an intercept of *α *= -6.6925 and coefficient of *β *= 0.5652. We would recommend these choices for the *de novo *classification of V5 sequences.

**Figure 8:**
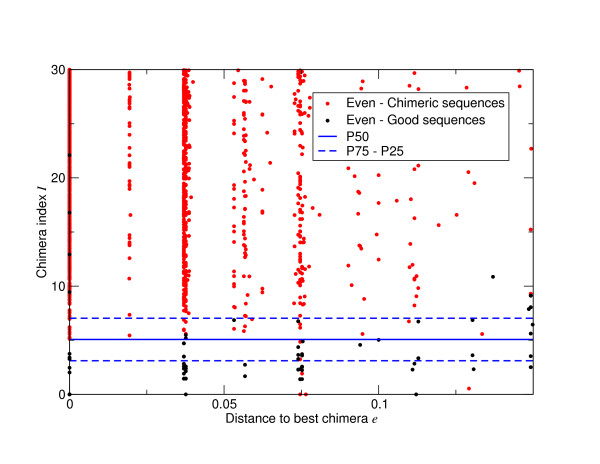
**Training logistic regression on denoised V2 'Even' data**. Good sequences are shown as black dots, chimeras red and reference sequences magenta. We used the three denoised V2 'Even' data sets, classified either good or chimeric by comparison with the references, and the reference sequences, all good, to train a one dimensional logistic regression on the 'chimera index' *I *using the R software package. An intercept, *α *= - 2.83542, and coefficient *β *= 0.55889 were obtained (highly significantly different from zero). The corresponding P25, P50 and P75 decision lines are shown (blue lines). The fit reduced the null deviance from 1371.81 on 5416 degrees of freedom to a residual deviance of 416.36 on 5415 degrees of freedom (AIC 420.36).

The results of the training on the three V2 'Even' sets of denoised sequences are shown in Figure 8. In this case with much more training data the logistic regression converges, generating both a P50 decision line and well defined uncertainties about that prediction given as the P25 and P75 lines, 25% and 75% probability of being chimeric respectively. Details of the regression are given in the caption of the figure. The results of applying this logistic regression to the three 'Uneven' validation data sets are given in Table 7. Here for each category of sequence, determined by direct comparison with the references, we give the number classified good or chimeric by the logistic regression with the P50 classification rule. For all data sets a very high sensitivity is achieved with around 99% of chimeras, both bimeras and trimeras, being removed. This is at a cost in false positive rates that varies from 10% to 15% of good sequences. We also note from Table 7 that two thirds of 'unclassified' sequences are flagged as chimeras. ChimeraSlayer can not achieve such high sensitivities, it misses some 15% of bimeras, and more of the trimeras, but the false positive rate is lower, at around 5% of good sequences (Table 8). We also trained the classifier on the *σ_s _*= 0.004 Titanium data in the same way obtaining *α *= -6.14268 and *β *= 0.40297. In this case we lacked a separate training and validation set but for the training data we identified 167/174 = 97.8% of the chimeras successfully at a cost of obtained 2/91 = 2.2% false positives.

**Table 7:** Chimera classification accuracies Perseus applied to the three denoised V2 'Uneven' data sets.

Dataset	Uneven1		Uneven2		Uneven3	
**Classification**	**Good**	**Chimeric**	**Good**	**Chimeric**	**Good**	**Chimeric**

Good	78 (83.0%)	16 (17.0%)	70 (90.9%)	7 (9.1%)	62 (82.7%)	13 (17.3%)
Bimera	7 (0.9%)	809 (99.1%)	9 (1.3%)	660 (98.7%)	10 (1.2%)	833 (98.8%)
Trimera	1 (1.2%)	80 (98.8%)	1 (1.4%)	70 (98.6%)	1 (1.2%)	81 (98.8%)
Quadramera	0 (0.0%)	1 (100.0%)	(0.0%)	2 (100.0%)	--	--
Unclassified	26 (19.7%)	106 (80.3%)	14 (35.0%)	26 (65.0%)	22 (41.5%)	31 (58.5%)

Each row gives a separate category of denoised sequence according to its true classification as 'Good', 'Bimera', 'Trimera', 'Quadramera' and 'Unclassified'. The columns are then split across data sets and give the number flagged as good or chimeric by classification with a logistic regression given a 50% probability cut-off.

**Table 8:** Chimera classification accuracies for ChimeraSlayer applied to the three denoised V2 'Uneven' data sets.

Dataset	Uneven1		Uneven2		Uneven3	
**Classification**	**Good**	**Chimeric**	**Good**	**Chimeric**	**Good**	**Chimeric**

Good	86 (91.5%)	8 (8.5%)	72 (93.5%)	5 (6.5%)	72 (96.0%)	3 (4.0%)
Bimera	125 (15.3%)	688 (84.3%)	98 (14.6%)	571 (85.4%)	108 (12.8%)	735 (87.2%)
Trimera	20 (24.7%)	61 (75.3%)	13 (18.3%)	58 (81.7%)	15 (18.3%)	67 (81.7%)
Quadramera	0(0.0%)	1 (100.0%)	(0.0%)	1 (50.0%)	--	--
Unclassified	55 (41.7%)	76 (57.6%)	15 (37.5%)	26 (62.5%)	27 (50.9%)	24 (45.3%)

Each row gives a separate category of denoised sequence according to its true classification as 'Good', 'Bimera', 'Trimera', 'Quadramera' and 'Unclassified'. The columns are then split across data sets and give the number flagged as good or chimeric by ChimeraSlayer at 50% bootstrap. Occasionally a sequence remained unclassified probably because there was no good NAST alignment. Consequently rows do not alway sum to 100%. The Broad Institute 'Gold' 16S rRNA sequences were used as references.

In practice, dedicated training data sets for each study may not be possible, although we would recommend it. If data is GS FLX V5 or V4-V5 Titanium then the two pairs of *α *and *β *values given above should work well for their corresponding data types. For V2 data, we would not recommend the values in the caption of Figure 8 because there is an implicit assumption of very high prior chimera probability generated from training on this atypical data set, reflected in the high value of *α *= -2.83542. The *β *values on all data sets are in the range of *β *= 0.5 and we have found that this value, paired with *α *= -7.5, performs well across a wide range of data sets.

## Conclusions

We have demonstrated that AmpliconNoise followed by Perseus has the sensitivity to remove the majority of errors from GS FLX and Titanium pyrosequenced amplicons and allow accurate estimates of OTU number. AmpliconNoise outperforms both agglomerative clusterers, SLP and the DeNoiser, both in terms of per base error rates and OTU construction but at a cost of increased computational complexity and no doubt in some cases, where some noise can be tolerated, these simpler heuristic approaches may be the best option. However, the results here suggest that both agglomerative approaches must be treated with caution, by not simply looking at OTU numbers as in previous evaluations [9,10], but rather their identity we have established that these are prone to over-clustering, removing a substantial fraction of the true diversity even at the 3% level.

Consequently, we believe that the AmpliconNoise-Perseus pipeline which is freely available as open source software [23] with all data on a dedicated website [24], will find a wide range of applications in microbial diversity estimation [8], and population genetics [2]. They could be critical to the success of large-scale publicly funded efforts to explore microbial diversity, such as the sequencing of human associated microbes being conducted in the Human Microbiome Project [25]. To facilitate their use we include programs for integrating their output into the QIIME 16S rRNA analysis pipeline [20].

We have shown the importance of considering both the effects of PCR and sequencing errors in studies of diversity based on 16S rRNA amplicons. This suggests the use of high fidelity polymerases to reduce per base PCR error rates. However, one of our most striking observations was just how variable chimera frequencies were in the test data sets. This must be due to PCR conditions, principally, cycle number, extension time, primer and template concentrations and polymerase type [26]. Therefore optimising the whole PCR process to minimise all types of errors is probably a better strategy than just focussing on the enzyme.

In addition, the principles outlined here: the rigorous validation of noise removal algorithms with test data; the use of EM algorithms to generate effective consensus sequences and remove noise from pyrosequenced amplicons; using sequence abundances in the classification of PCR chimeras; and the treatment of the latter as a supervised learning problem; will provide the basis for further algorithm development in this field and contribute to the maturation of next generation sequencing as a quantitative technique for the analysis of PCR amplicon diversity. These principles will be equally applicable to other pyrosequencing platforms, for example the Illumina HiSeq 2000 which is capable of generating orders of magnitude more reads per run [27,28]. They should even hold for the third generation of sequencing technologies that will target individual molecules [29]. As ever larger amounts of sequence data are generated the question of how to distinguish true diversity from noise will become ever more important.

The Titanium full length clones sequences have been submitted to GenBank with accession numbers HQ462473-560. The Titanium pyrosequencing reads have been submitted to the Short Read Archive with accession SRP003773.

## Authors' contributions

CQ designed the algorithms, wrote software, performed analyses and wrote the paper. AL contributed to the analyses and writing of the paper. RJD performed experiments and helped write the paper. PT designed and performed the experiments and helped write the paper.
